# A viscoelastic anisotropic hyperelastic constitutive model of the human cornea

**DOI:** 10.1007/s10237-017-0942-2

**Published:** 2017-08-05

**Authors:** Charles Whitford, Natalia V. Movchan, Harald Studer, Ahmed Elsheikh

**Affiliations:** 1 School of Engineering, University of Liverpool, The Quadrangle, Brownlow Hill, Liverpool, L69 3GH UK; 2 Department of Mathematical Sciences, University of Liverpool, Mathematical Sciences Building, Liverpool, L69 7ZL UK; 3 Integrated Scientific Services AG, Biel, Switzerland; 4 National Institute for Health Research (NIHR) Biomedical Research Centre at Moorfields Eye Hospital NHS Foundation Trust and UCL Institute of Ophthalmology, London, UK

**Keywords:** Cornea, Biomechanics, Viscoelastic, Numerical, Anisotropic, Constitutive

## Abstract

A constitutive model based on the continuum mechanics theory has been developed which represents interlamellar cohesion, regional variation of collagen fibril density, 3D anisotropy and both age-related viscoelastic and hyperelastic stiffening behaviour of the human cornea. Experimental data gathered from a number of previous studies on 48 *ex vivo* human cornea (inflation and shear tests) enabled calibration of the constitutive model by numerical analysis. Wide-angle X-ray scattering and electron microscopy provided measured data which quantify microstructural arrangements associated with stiffness. The present study measures stiffness parallel to the lamellae of the cornea which approximately doubles with an increase in strain rate from 0.5 to 5%/min, while the underlying stromal matrix provides a stiffness 2–3 orders of magnitude lower than the lamellae. The model has been simultaneously calibrated to within 3% error across three age groups ranging from 50 to 95 years and three strain rates across the two loading scenarios. Age and strain-rate-dependent material coefficients allow numerical simulation under varying loading scenarios for an individual patient with material stiffness approximated by their age. This present study addresses a significant gap in numerical representation of the cornea and has great potential in daily clinical practice for the planning and optimisation of corrective procedures and in preclinical optimisation of diagnostic procedures.

## Introduction

The ocular vessel consists of the cornea, sclera and corneoscleral limbal junction. The vessel protects the internal contents of the eye and maintains the eye’s general shape which is necessary for clear vision. The transparent cornea, at the anterior segment of the eye, provides two-thirds of the eye’s optical power (Fatt 1978), and this contribution is primarily determined by the cornea’s external topography, clarity and refractive index relative to the external environment.

The topography of the cornea is determined by the balanced state between the forces acting upon it and its mechanical stiffness, which is defined by cornea’s geometry, thickness and the material stiffness. While the geometry and thickness, and their contribution to overall mechanical stiffness, are easy to determine, the material stiffness is much more difficult to quantify as it is dependent on the microstructure of the stroma, the main load carrying layer of the cornea. The stroma is comprised of over 200 lamellae (Freegard 1997; Oyster 1999), each of which formed of a proteoglycan-rich matrix containing tightly packed and ordered collagen fibrils. The density and orientation of collagen fibrils in the stroma are the primary factors affecting the material stiffness and hence the overall mechanical stiffness of the cornea (Jue and Maurice 1991; Newton and Meek 1998; Boote et al. 2003, 2009). Wide-angle X-ray scattering (WAXS) has been extensively used to detail the 2D anisotropic arrangement of collagen fibrils in the human cornea (Aghamohammadzadeh et al. 2004; Meek and Boote 2004; Boote et al. 2006). Further, the 3D organisation of fibrils was observed by Komai and Ushiki (1991) using electron microscopy where the arrangement of lamellae and inter-lamellae fibrils was observed. Whitford et al. (2015) analysed the data within these studies and extracted relationships defining the regional variation of collagen fibril density and anisotropy across the corneal surface.

To date, there have been a significant number of studies which have progressed the numerical representation of the cornea in its quasi-static state. These have included Alastrue et al. (2006), Pandolfi and Manganiello (2006), Pandolfi and Holzapfel (2008), Pinsky et al. (2005), Petsche and Pinsky (2013), Studer et al. (2010), Grytz and Meschke (2009, 2010), Nguyen and Boyce (2011) and Whitford et al. (2015). Further, dynamic (non-static) behaviour of the cornea has been modelled in various studies. Glass et al. (2008) developed an isotropic, homogeneous, analytical model describing the effect of viscosity and elasticity on hysteresis in the human cornea. Perez et al. (2013) developed a viscoelastic model of the eye that was limited to linear-elastic, isotropic representation of porcine eyes with a homogeneous corneal representation. Kling et al. (2014) considered an isotropic, linear, viscoelastic corneal model within a multi-physics simulation of air-puff tonometry. Others (Boyce et al. 2007; Cui et al. 2015; Nguyen et al. 2008; Su et al. 2015) developed viscoelastic constitutive models which were used to describe the behaviour of bovine cornea based on the results of strip extensometry.

To the author’s knowledge, this is the first study that combines the complex anisotropic representation, shear stiffness and regional variation of fibril density of the human cornea with its viscoelastic behaviour. The present study further attempts to calibrate the proposed model with existing *ex vivo* human data. The research builds on a recent study by Whitford et al. (2015) that introduced the representation of regional variation of collagen fibril density and proposes a constitutive model that decomposes the stress–strain behaviour into four components representing: (1) dilation, (2) isotropic matrix distortion response to both tension and compression, (3) anistropic and regional variation of collagen fibrils and (4) the time-dependent component which represents the nonlinear, strain- rate dependence of behaviour as a departure from the equilibrium response.

## Methods and materials

### Constitutive model

The nonlinear anisotropic incompressible material behaviour of the corneal stroma can be numerically represented using the Helmholtz free-energy function:

1 $$\begin{aligned} \psi =\psi \left[ \mathbf {C,A,B}\right] , \end{aligned}$$

where $\mathbf {C}$ is the right Cauchy–Green deformation tensor calculated from $\mathbf {C}=\mathbf {F}^T\mathbf {F}$ with $\mathbf {F}$ being the deformation gradient, a second-order tensor representing the gradient of the mapping function and relating the current configuration of a continuum to its reference configuration. $\mathbf {A}=\mathbf {a}\otimes \mathbf {a}$ and $\mathbf {B}=\mathbf {b}\otimes \mathbf {b}$ are anisotropic tensors, based on vectors $\mathbf {a}$ and $\mathbf {b}$ which define single discretised directions of anisotropy. Similar to a procedure presented earlier (Studer et al. 2010), an isochoric split is performed on the energy density function to separate the responses to a volume-changing dilation and a volume-preserving distortion:

2 $$\begin{aligned} \psi =U\left[ J\right] +\bar{\psi }\left[ \bar{\mathbf {C},A,B}\right] , \end{aligned}$$

where $\bar{\mathbf {C}}$ is the distortion component of the right Cauchy–Green deformation tensor defined from $\mathbf {C}=\left( J^{2/3}\mathbf {I}\right) \bar{\mathbf {C}}=J^{2/3}\bar{\mathbf {C}}$ where $\mathbf {I}$ represents the unit tensor, $J=\det \left( \bar{\mathbf {F}}\right)$, $\mathbf {F}=\left( J^{1/3}\mathbf {I}\right) \bar{\mathbf {F}}$ and $\bar{\mathbf {F}}$ defines the deformation gradient associated with distortion. Further explanation of these concepts is provided by Holzapfel (2000) and others. In order to provide separate representations of the matrix’ and fibrils’ contributions to mechanical behaviour, a second split of the free-energy function is performed:

3 $$\begin{aligned} \psi =U\left[ J\right] +\bar{\psi }_\mathrm{m}\left[ \bar{\mathbf {C}}\right] +\bar{\psi }_\mathrm{f}\left[ \bar{\mathbf {C},A,B}\right] , \end{aligned}$$

As in Whitford et al. (2015) and other studies, the dilation constituent, *U*[*J*], from Eq. 3 is given by:

4 $$\begin{aligned} U\left[ J\right] =\frac{1}{D}\left( J-1\right) ^2, \end{aligned}$$

where *D* is the material coefficient describing volumetric compressibility. Also in the neo-Hookean formulation, the constituent equation to represent the matrix stiffness is given by:

5 $$\begin{aligned} \bar{\psi }_\mathrm{m}\left[ \bar{I}_1\right] =C_{10}\left( \bar{I}_1-3\right) , \end{aligned}$$

where the distortion component of the right Cauchy–Green deformation tensor, $\bar{\mathbf {C}}$, was replaced by its first strain invariant; $\bar{I}_1=\hbox {tr}\bar{\mathbf {C}}$, and $C_{10}$ is a material constant.

Since in this equation $\mathbf {A}$ and $\mathbf {B}$ are second order tensors and each can only represent a single direction of anisotropy, an adaptation is required to enable consideration of a multi-directional fibril orientation. Pinsky et al. (2005) presented a numerical method to describe the angular distribution of collagen fibrils in the corneal and limbal stroma obtained from WAXS studies (Aghamohammadzadeh et al. 2004). This method was later modified by Studer et al. (2010). The coordinate system adopted is presented in Whitford et al. (2015). Also from Whitford et al. (2015) the free-energy function describing the fibril response is given by:

6 $$\begin{aligned} \begin{aligned}&\bar{\psi }_\mathrm{f}\left[ {\bar{\mathbf {C}},A,B}\right] \\&\quad =\zeta \frac{1}{\pi }\int _0^\pi \chi \bigg (\bar{\psi }_{f,\mathrm{lamellae}}\left[ {\bar{\mathbf {C}},A}\right] +\bar{\psi }_{f,\mathrm{ILC}}\left[ {\bar{\mathbf {C}},B}\right] \bigg )\mathrm{d}\theta _L, \end{aligned} \end{aligned}$$

where $\zeta$ represents the global density function and $\chi$ represents the local density function, defining the anisotropic distribution of the fibrils. The lamellae and interlamellae cohesion (ILC) fibril contributions to the constituent equation were based on the polynomial Ogden law, modified by Markert et al. (2005) to include one direction of anisotropy which has been shown to accurately represent the 3D behaviour of the microstructure (Whitford et al. 2015). They were therefore rewritten as:

7 $$\begin{aligned} \begin{aligned}&\bar{\psi }_{f,\mathrm{lamellae}}\left[ \bar{I}_4\right] =\frac{\mu _1}{\gamma _1}\left( \bar{I}_4^{\frac{\gamma _1}{2}}-1\right) -\mu _1\ln \bar{I}_4^{\frac{1}{2}},\\&\bar{\psi }_{f,\mathrm{ILC}}\left[ \bar{I}_6\right] =\frac{\mu _2}{\gamma _2}\left( \bar{I}_6^{\frac{\gamma _2}{2}}-1\right) -\mu _2\ln \bar{I}_6^{\frac{1}{2}}, \end{aligned} \end{aligned}$$

where $\bar{\mathbf {C}}$, $\mathbf {A}$ and $\mathbf {B}$ in Eq. 6 are replaced by the invariants $\bar{I}_4=\bar{\mathbf {C}}:\left( \mathbf {a}\otimes \mathbf {a}\right)$, $\bar{I}_6=\bar{\mathbf {C}}:\left( \mathbf {b}\otimes \mathbf {b}\right)$ and material parameters $\mu _{1,2}$ (polynomial coefficient relating to stiffness) and $\gamma _{1,2}$ (governing nonlinearity).

The condition where the fibril constituent of Eq. 3 is only activated where tension is applied, $\lambda _{4,6}=I_{4,6}^{\frac{1}{2}}>1$, as it is considered that only the matrix carries compressive forces.

Numerical parameters $\zeta$ and $\chi$ represent the global and local distributions of collagen fibrils, respectively. The derivation and definitions of these parameters can be found in Whitford et al. (2015). It is likely that there is through-thickness inhomogeneity which would result in variation of mechanical properties through the thickness of the stroma. However, a lack of data on microstructure mapping out of tangential plane and experimental data which homogenised through-thickness behaviour led to consistent through-thickness representation of biomechanical properties.

To accommodate rate dependency within the model, the response of the material becomes a function of time, $t\in \left[ 0,T\right]$, where reference time $t=0$ relates to the reference configuration, $\varOmega _0$. Viscoelastic effects are described using the concept of internal variables. These variables are not accessible to direct observation; they describe the internal structure of the material associated with the irreversible (dissipative) effects (Holzapfel et al. 2000). Viscoelastic behaviour is modelled by $m \ge 1$ relaxation processes with corresponding relaxation times, $\tau _\alpha \in \left[ 0,\infty \right]$, $\alpha =1,\ldots ,m$ $\left( m \ge 1\right)$, describing the rate of decay of the stress. These material variables vanish at the equilibrium state, which does not depend on time. The internal variables are denoted by $\varGamma _\alpha ,\, \alpha =1,\ldots ,m$.

Mathematically, the adaptation of the model to represent viscoelastic response could be performed prior to the isochoric split, or split between matrix and fibril definitions, therefore accommodating viscoelastic behaviour of the dilation and/or the matrix within the model. However, the matrix and dilation contributions to stiffness have been shown to have relatively less contribution to stiffness than fibril behaviour (Whitford et al. 2015). Holzapfel and Gasser (2001) presented a model where the viscoelastic behaviour was a function of the distortion component of the free energy after the isochoric split had been performed. That model is modified here, and the dissipative potentials are introduced providing the viscoelastic constituent as a function of the fibril constituent, $\sum _{\alpha =1}^m\bar{\psi }^\infty _{f\,\alpha }\left[ \bar{\mathbf {C}},\mathbf {A},\mathbf {B},\bar{\varGamma }_\alpha \right]$. The tangential and out-of-tangential fibril constituents of the model being functions of the fourth and sixth strain invariants, respectively, lead to $\sum _{\alpha =1}^m \, \sum _{a=4,6}\bar{\psi }^\infty _{f\,\alpha \, a}\left[ \bar{\mathbf {C}},\bar{I}_a,\bar{\varGamma }_\alpha \right]$, and the free-energy function from Eq. 3 becomes:

8 $$\begin{aligned} \psi =U^\infty \left[ J\right] +\bar{\psi }^\infty _\mathrm{m}\left[ \bar{I}_1\right] + \bar{\psi }^\infty _\mathrm{f}\left[ \bar{I}_{4,6} \right] +\sum _{\alpha =1}^m \bar{\psi }^\infty _{f\,\alpha }\left[ \bar{\mathbf {C}},\bar{I}_{4,6},\bar{\varGamma }_\alpha \right] \end{aligned}$$

At this stage, the symmetric second Piola–Kirchhoff stress tensor can be written describing the equilibrium stress response of the material:

9 $$\begin{aligned} \mathbf {S}^{\infty }=\mathbf {S}_\mathrm{dil}^{\infty }+\mathbf {S}_\mathrm{m}^{\infty }+\mathbf {S}_\mathrm{f}^{\infty }. \end{aligned}$$

The three contributions to the constitutive model, $\mathbf {S}^{\infty }_\mathrm{dil}$, $\mathbf {S}^{\infty }_\mathrm{m}$ and $\mathbf {S}^{\infty }_\mathrm{f}$, describe the dilation, and the isotropic and anisotropic distortion responses of the matrix and fibres, respectively. These are given by:

10 $$\begin{aligned} \mathbf {S}^{\infty }_\mathrm{dil}=2\frac{\partial U^\infty }{\partial \mathbf {C}}, \,\, \mathbf {S}^{\infty }_\mathrm{m}=2\frac{\partial \bar{\psi }^\infty _\mathrm{m}}{\partial \mathbf {C}}, \,\, \mathbf {S}^{\infty }_\mathrm{f}=2\frac{\partial \bar{\psi }^\infty _\mathrm{f}}{\partial \mathbf {C}} \end{aligned}$$

From Holzapfel and Gasser (2001) the rate dependency is expressed as an additional component to the constitutive equation at time $t_{n+1}$ and an adaptation of the stress function is required where the non-equilibrium stresses, $\mathbf {Q}_\alpha =J^{-2/3}\mathbb {P}:\hat{\mathbf {Q}}_\alpha$ where the fourth-order projection tensor, $\mathbb {P}$, is given by:

11 $$\begin{aligned}&\mathbb {P}=\mathbb {I}-\mathbf {C}^{-1}\otimes \left( \mathbf {C}/3\right) , \,\,\,\, \mathbb {I}_{IJKL}= \Big (\delta _{IK}\delta _{JL}+\delta _{IJ}\delta _{KL}\Big )/2 \nonumber \\\end{aligned}$$

12 $$\begin{aligned}&\hat{\mathbf {Q}}_\alpha =2\frac{\partial \bar{\psi }_{f \, \alpha }\left[ \bar{\mathbf {C}}, \mathbf {A}, \mathbf {B} \right] }{\partial \bar{\mathbf {C}}} \end{aligned}$$

The internal dissipation is defined as: ${\varvec{\mathcal {D}}}_\mathrm{int} =\sum _{\alpha =1}^m \mathbf {Q}_\alpha : \dot{\bar{\varGamma }}_\alpha / 2 \ge 0$. As the dissipation vanishes at equilibrium $\left( t\rightarrow \infty \right)$

13 $$\begin{aligned} \mathbf {Q}_\alpha = \left. -2 \frac{\partial \bar{\psi }_{f\alpha }}{\partial \bar{\varGamma }_\alpha }\right| _{t \rightarrow \infty }=\mathbf {0}, \,\,\,\, \alpha =1,\ldots ,m \end{aligned}$$

and Eq. 9 becomes:

14 $$\begin{aligned} \mathbf {S}_{n+1}=&\left( \mathbf {S}^{\infty }_\mathrm{dil}+\mathbf {S}^{\infty }_\mathrm{m} +\mathbf {S}^{\infty }_\mathrm{f}+\sum \limits _{\alpha =1}^m \mathbf {Q}_\alpha \right) _{n+1} \end{aligned}$$

where the non-equilibrium stresses are defined by:

15 $$\begin{aligned} \left( \mathbf {Q}_{\alpha }\right) _{n+1}= \left( {\varvec{\mathcal {H}}}_{\alpha }\right) _{n} + \beta ^\infty _\alpha \exp \left( \xi _\alpha \right) \left( \mathbf {S}^{\infty }_\mathrm{f}\right) _{n+1}, \,\,\, \alpha =1,\ldots ,m \end{aligned}$$

and the definition of the history term, $\left( {\varvec{\mathcal {H}}}_{\alpha }\right) _{n}, \, \alpha =1,\ldots ,m$, is modified from Holzapfel and Gasser (2001) providing

16 $$\begin{aligned} \begin{aligned}&\left( {\varvec{\mathcal {H}}}_{\alpha }\right) _{n}=\exp \left( \xi _\alpha \right) \left( \exp \left( \xi _\alpha \right) \left( \mathbf {Q}_{\alpha }\right) _{n} - \beta ^{\infty }_{\alpha } \left( \mathbf {S}^{\infty }_\mathrm{f}\right) _{n} \right) , \\&\xi _\alpha =-\frac{\Delta t}{2 \tau _{\alpha }} \end{aligned} \end{aligned}$$

$\beta _\alpha ^\infty \in \left[ 0,\infty \right]$ and $\tau _\alpha \in \left[ 0,\infty \right] , \, \alpha =1,\ldots ,m$ are non-dimensional and time-dimensional free-energy factors, respectively. These remain to be defined. For mathematical purposes, an approximation is made that the viscoelastic stress of the reference configuration $\mathbf {Q}^{0+}_{\alpha }=\mathbf {0}$. The accuracy of this approximation relates to the implementation of the constitutive model and is discussed later in the study. The stiffness tensor at $t_{n+1}$ can similarly be written as:

17 $$\begin{aligned} \mathbf {D}_{n+1}=\left( \mathbf {D}^\infty _\mathrm{dil} + \mathbf {D}^\infty _\mathrm{m} + \mathbf {D}^\infty _\mathrm{f} + \sum \limits _{\alpha =1}^m \mathbf {D}^\alpha _\mathrm{vis} \right) _{n+1} \end{aligned}$$

where

18 $$\begin{aligned} \mathbf {D}^{\infty }_\mathrm{dil} =2\frac{\partial \mathbf {S}^\infty _\mathrm{dil}}{\partial \mathbf {C}}, \,\,\,\,\,\, \mathbf {D}^{\infty }_\mathrm{m} =2\frac{\partial \mathbf {S}^\infty _\mathrm{m}}{\partial \mathbf {C}}, \,\,\,\,\,\, \mathbf {D}^{\infty }_\mathrm{f} =2\frac{\partial \mathbf {S}^\infty _\mathrm{f}}{\partial \mathbf {C}} \end{aligned}$$

and

19 $$\begin{aligned} \left( \mathbf {D}_\mathrm{vis}^\alpha \right) _{n+1} = \delta _{\alpha } \left( \mathbf {D}^{\infty }_\mathrm{f}\right) _{n+1}, \,\, \delta _{\alpha } = \beta ^{\infty }_{\alpha }\exp \left( \xi _\alpha \right) , \,\, \alpha =1,\ldots ,m \end{aligned}$$

**Fig. 1:**
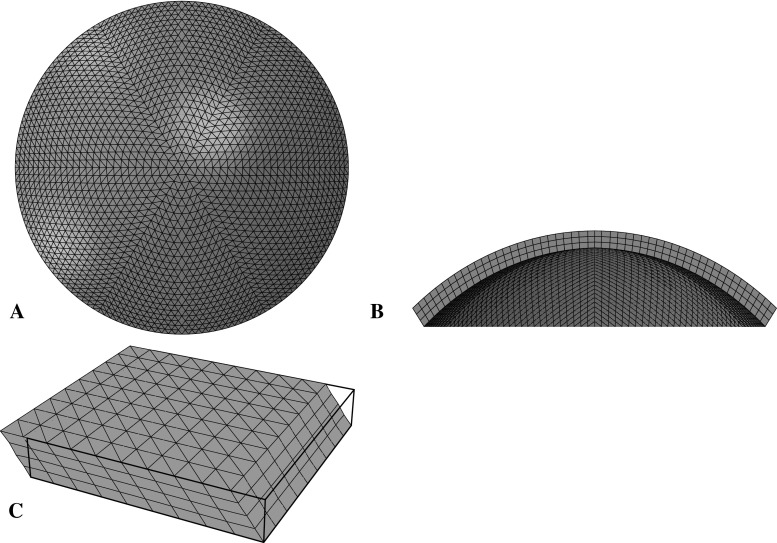
Finite element models (FEM) used during analysis: **a** anterior and **b** section views of the human cornea model used to simulate inflation tests, and **c** the model used to simulate shear tests (deformed and, superimposed, undeformed geometries)

### Implementation of numerical simulation

Numerical simulations have been conducted using finite element analysis (FEA). Geometric modelling was performed using bespoke software that provides geometry, which can be imported into finite element solvers as an orphan mesh. Finite element solver Abaqus/Standard 6.13 (Dassault Systemes Simulia Corp., Rhode Island, USA) was used. Abaqus is well known for its ability to analyse nonlinear problems. However, its ability to provide state-of-the-art representation of biological material properties, and both regional and local variation of these properties, is limited. Thus, Abaqus was used in conjunction with bespoke subroutines (SDVINI & UMAT) written in FORTRAN to implement the constitutive model described above.

The integral of Eq. 6 was discretised into steps of one degree by $\frac{1}{\pi }\int _0^{\pi }{}\mathrm{d}\theta _L\rightarrow \frac{1}{180}\sum _{i=1}^{180}\theta _{L,i}$, where $\theta _{L,i}$ defines the orientation of the one hundred and eighty directions of anisotropy per integration point within the model. The assumption that the fibril constituent of the model only provided tensile stiffness was adopted throughout all behaviour stages.

Subroutine SDVINI was used to provide initial, reference-configuration and location-based conditions such as fibril density representation. These location-based properties are defined individually for each integration point.

These are implemented into the numerical simulation using the UMAT subroutine as demonstrated in the Abaqus User Subroutines Reference Guide (Abaqus 2013). UMAT is also used to define current-configuration properties such as anisotropy.

Models were generated using fifteen-noded, solid, hybrid, quadratic elements (Abaqus, C3D15H). The cornea inflation models consisted of three layers and 26 rings of elements. The shape of elements, and their arrangement, was chosen to provide uniform element sizes and consistent approximation of geometry. Trials of these models indicated analysis periods of 5–10 mins depending on variations in materials definitions. Thickness values were assumed to be rotationally symmetric; central corneal thickness (CCT) of 545 $\upmu$m was used as the average value reported from a number of previous studies (Francis et al. 2007). Peripheral corneal thickness (PCT), measured normal to the anterior surface, was 150 $\upmu$m greater than the CCT, consistent with Gullstrand’s No.1 schematic eye (Bennet and Rabbetts 1989). A shape factor of 0.82 was applied to the anterior surface of the model to provide a representative non-spherical surface (Douthwaite et al. 1999). The near incompressibility of the corneal stroma is represented by hybrid elements which provide volume controls within the solver (Abaqus Theory Manual), and the constant *D* (Eq. 4) was set to the low value of $10^{-5}$, indicating close to incompressible behaviour. Similar to Pandolfi and Holzapfel (2008), the remaining dilation term of Eq. 8 becomes purely mathematically motivated. The arrangement of elements, three layers and 24 rings, (Fig. 1), was chosen by increasing the number of element layers and rings by assessing the convergence of the solution in a mesh-density sensitivity study. The number of element rings was controlled by the number of element layers such that the aspect ratio of the elements approached 1. The number of layers, and therefore rings, was increased until the difference of apical deformation in the subsequent simulation with further refinement became less than 0.1%. C3D15H elements contain nine integration points. It was judged that the number of elements provided good refinement regarding the regional variation of material properties, which were individually characterised for each integration point.

**Fig. 2:**
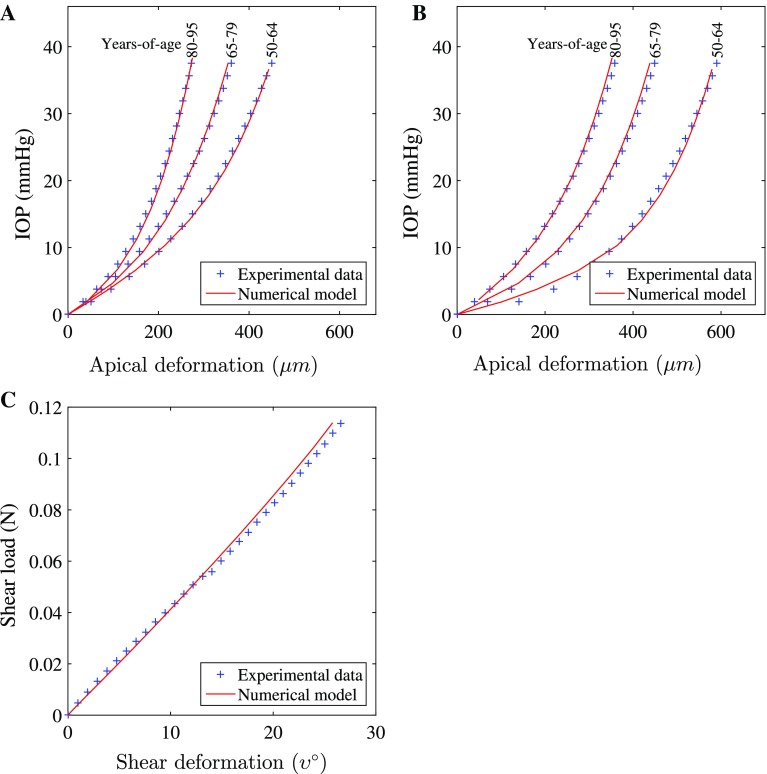
Characteristic experimental data and results of numerical simulation: **a** corneal inflation, 37.5 mmHg/min; **b** corneal inflation, 3.75 mmHg/min; **c** corneal shear, 10%/min deformation

### Derivation of material properties

Characteristic experimental data have been obtained from 48 fresh human donor corneas. Data include 36 corneas tested under inflation and 12 corneas tested under shear. The corneas tested under inflation were divided into two groups: 23 corneas tested with 37.5 mmHg/min pressure rate and 13 corneas tested with 3.75 mmHg/min rate (Elsheikh et al. 2007). The age range of the two groups was 51–95 $\,(77.6 \pm 13.2)$ and 50–95 $\,(75.7 \pm 14.2)$ years, respectively. Within each group, the corneas were divided into three age subgroups: 50–64, 65–79 and 80–95 years. The number of corneas tested under 37.5 mmHg/min was 4, 6 and 13 within the three age subgroups, respectively. The corresponding numbers tested under 3.75 mmHg/min were 4, 4 and 5. Twelve human donor corneas, aged between 61 and 74 years $(67.7 \pm 5.8)$, were tested to determine the behaviour of stromal tissue under surface shear at a shear deformation rate of $10\%$/min (with respect to the tissue’s thickness) (Elsheikh et al. 2009). Shear tests do not generate strains parallel to the tangent plane, allowing the isolation of out-of-tangential behaviour during numerical analysis. In contrast, inflation generates multi-axis strain, including relatively large tangential strains. The isolation of material behaviour through multi-objective experimental fitting was utilised in Whitford et al. (2015) and is again utilised in this study. In addition to this isolation of anisotropic stiffness calibration, simultaneous fitting to 3 different loading rates allowed the calibration of viscoelastic parameters.

The external parameters $\left( \zeta , \chi \right)$ describing the local and global variation in fibril distribution and the internal parameters $\left( C_{10}, D \right)$ which describe the stiffness of the matrix and the volume change are unaffected by the introduction of the internal variables, relating to the viscoelastic behaviour. These values therefore remain as derived in Whitford et al. (2015). However, the internal parameters $\left( \mu _{1,2}, \gamma _{1,2} \right)$ are intrinsically combined with the viscoelastic parameters in the partial differential equations of the viscoelastic behaviour. Further, in earlier studies describing the anisotropic distribution of collagen fibrils, for example Pinsky et al. (2005), Studer et al. (2010), Whitford et al. (2015), the material parameters were derived to define the hyperelastic response at a non-equilibrium state. The inclusion of the viscoelastic term to the fibril representation requires that parameters $\mu _{1,2}, \gamma _{1,2}$ describing the fibril response are redefined such that they are intended to represent the equilibrium behaviour.

The parameters which remain to be determined $(\mu _{1,2}, \gamma _{1,2},$ $\beta _{\alpha }, \tau _{\alpha }: \alpha =1,\ldots ,m)$ were derived using a multi-objective inverse analysis procedure. This optimisation process utilised the optimisation software HEEDS (Red Cedar Technology, Michigan, USA). Within HEEDS, the SHERPA algorithm was utilised. This algorithm incorporates Monte Carlo sampling; this ensured that the analysis did not stop at local minima and that the resulting values were unique and robust. The objectives were to reduce the root-mean-square (RMS) errors between the characteristic experimental results for corneal shear and inflation and their respective numerical simulations. In the study by Whitford et al. (2015), the parameters defining shear behaviour could be derived independently as the parameters defining tangential stiffness had no influence on this behaviour. However, due to the necessary approximation that the viscoelastic behaviour of the ILC fibrils is the same as the lamellae fibrils, the viscoelastic parameters for both family of fibrils require simultaneous derivation. The constitutive model above has been expressed for multiple orders of viscoelastic behaviour which can be represented through the use of the $\alpha$ term $\left( \alpha =1,\ldots ,m\right)$. The derivation process for material parameters included trials to determine the appropriate value for *m*.

## Results

Numerical simulations were fitted to characteristic experimental data (Fig. 2). Initial trials were conducted utilising a first-order viscoelastic model during which a root-mean-square error (RMS) for the age group 80–95 years of $4\%$ of the total deformation simulated (200–550 $\upmu$m) was achieved. However, with decreasing age, the RMS increased to $5\%$ for age group 50–65; the RMS for shear inflation was $3\%$. The fitting trend between age groups resulted in overestimation of displacement at low IOP and underestimation at higher IOP for the 50–65 age groups with a reversal of this trend when representing the 80–95 age group. Inverse analysis trials to derive material parameters were also conducted on a second-order viscoelastic model. For these separate trials, the RMS for all age groups and loading rates of inflation simulations and shear was less than $3\%$. The greatest error of $3\%$ was observed in the youngest age group, with less than $1.5\%$ obtained for the oldest group. Second-order corneal inflation models ran in approximately 5 min.

**Table 1:** Numerical parameters (*D*, $C_{10}$, $\mu _2$, $\gamma _2$) derived for the constitutive model describing the anisotropic, viscoelastic and hyperelastic corneal behaviour. These 4 parameters were found to be constant across 3 different age groups from 50 to 95 years of age

Parameter	Value
*D* (kPa)	0.01
$C_{10}$ (kPa)	9
$\mu _2$ (kPa)	3850
$\gamma _2$ (–)	$7.42\times 10^{-6}$

**Fig. 3:**
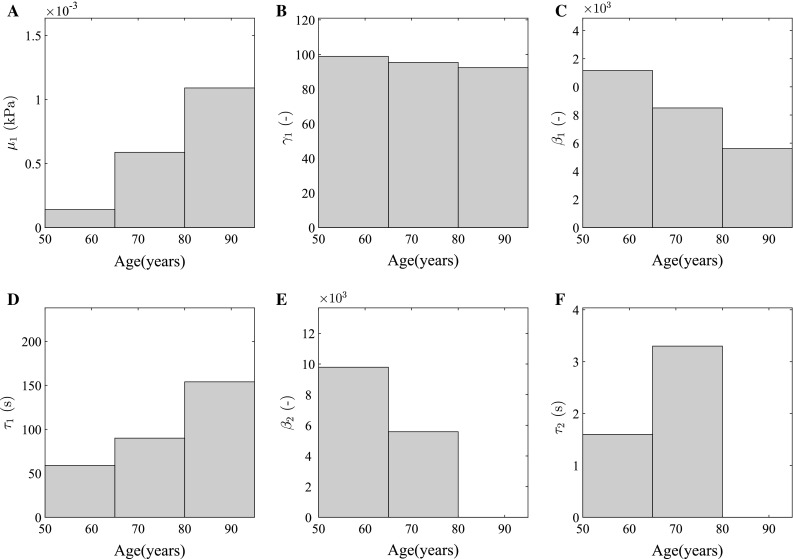
Numerical parameters [$\mu _{1}$ (**a**), $\gamma _{1}$ (**b**), $\beta _1$ (**c**), $\tau _1$ (**d**), $\beta _2$ (**e**) and $\tau _2$ (**f**)] derived for the constitutive model describing the anisotropic, viscoelastic and hyperelastic corneal behaviour from 50 to 95 years of age. The bar charts provide the discrete values derived for the best fit with each age group. Other numerical parameters are constant with age and are presented in Table 1

Numerical parameters found to vary with changes in age are provided in Fig. 3

**Fig. 4:**
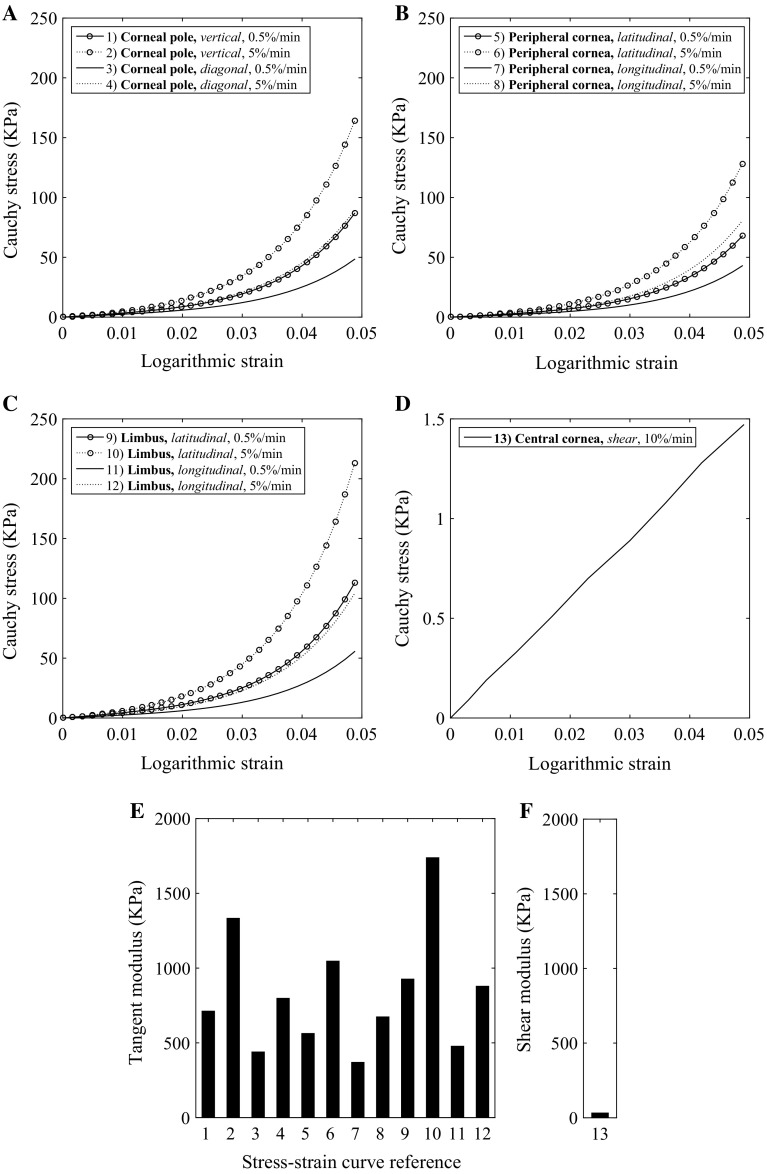
Material stiffness relationships representing characteristic behaviour of a 87-year-old: **a**–**c** regional and directional specific stress–strain behaviour obtained parallel to the tangent plane; **d** stress–strain relationship representing shear behaviour [note the different scale on the stress axis compared with plots (**a**–**c**)]; **e** tangent modulus for stress–strain relationships 1–12 (numbering relates to the numbered stress–strain relationships in **a**–**d**; **f** shear modulus. Values represent the stiffness at 0.02 logarithmic strain

Parameters of the proposed model have been simultaneously determined to represent characteristic shear and inflation responses across 3 different loading rates and for 3 age groups (Table 1; Fig. 3). As previously described, during the multiple iterations of analysis, both $\gamma _2$ and $\mu _2$, governing the equilibrium behaviour of ILC fibrils, were free to optimise. However, for each of these variables, respectively, the output of the procedures consistently provided values within 0.05% of each other across each age group. Due to this nonsignificant difference, results have been provided based on the mean of these values and are therefore constant with age.

Stiffness varies directionally and by location across the entire cornea as previously described (Whitford et al. 2015). Figure 4 provides the stiffness in the form of stress–strain relationships and measurements of tangent modulus at selected discrete locations and directions across the cornea and varying strain rates. Figure 4a–c provides the hyperelastic stress–strain relationships on the tangential plane of the cornea at 0.5 and 5%/min strain. Consistently, the higher strain rate results in higher stiffness when compared to the same location and direction. The greatest stiffness is observed circumferentially at the limbus which relates to the arrangement and density of the fibrils. Of the stiffness relationships presented, the lowest stiffness is in the diagonal direction at the corneal pole. Figure 4d provides the linear stress–strain relationship under shear at 10%/min, where this strain rate relates to translational motion of the top surface of the cornea in relation to the lower surface with respect to its thickness. Figure 4e highlights the tangent modulus at 2% strain, and Fig. 4f presents the shear stiffness. From Fig. 4e, f, it is clear that the shear stiffness is significantly less than tangential stiffness at 31.5 kPa compared to the range presented across the cornea for tangential stiffness, 370–1738 kPa. In the range of 0–0.05, strain tangent modulus (stiffness) is almost double at a strain rate of 5%/min with respect to 0.5%/min for the respective location and direction.

## Discussion

Within this study, a numerical representation of corneal microstructure has been developed within the continuum framework and applied to FEA. The model was applied to an extensive experimental database to obtain numerical relationships which describe regional variation of collagen density and anisotropy; the lamellae and ILC stiffness; the stiffness variation with age; strain-rate-dependent viscoelastic behaviour; and the viscoelastic variation with age (density and anisotropy being described in earlier studies such as Whitford et al. 2015). As in Whitford et al. (2015), density and anisotropic distribution of fibrils could not be observed or modelled with respect to age. It was suggested in that earlier study that variation in stiffness with age could be a function of fibril behaviour, not arrangement. This hypothesis is expanded in the current study due to the ability of the model to accurately and simultaneously represent age-related stiffening and age-related viscoelasticity changes without the need to change microstructural arrangement representation.

The results of calibrating the new constitutive model which has been presented here provide the relationship between viscoelastic behaviour and age. Previous presentation of the cornea’s strain-rate- dependent stiffness has not been able to isolate the age-related stiffening behaviour from the age-related viscoelastic changes. In the new constitutive model, parameters defining the viscoelastic behaviour, $\beta$ and $\tau$, define the initial viscoelastic behaviour of the fibrils and the rate of decay of the viscoelasticity. It has been shown that both the rate of decay and initial behaviour increase with age. During trials, it was found that a second-order viscoelastic model was most appropriate particularly in the 50–64 years of age group as a first order model was not able to accommodate the change in the second derivative of the load–deformation curves between the slow and faster loading rates of the inflation data. This change in the second derivative was accurately modelled with the second-order model. However, the second contribution to viscoelasticity tended to zero in the age group 80–95 years of age.

The model which has been presented here introduces a viscoelastic constituent to the model presented by Whitford et al. (2015). That model attributed the regional and anisotropic distribution of stiffness to fibril density and arrangement. In this model, the viscoelasticity decays with time $(t\rightarrow \infty )$, with its initial contribution being proportional to the behaviour of the fibrils. Although the model disregards the contributions from the matrix components to the viscoelastic behaviour, the overall behaviour is not affected significantly, since these components have been shown to have relatively low stiffness (Whitford et al. 2015).

These findings may be of increased importance where the application of high-speed techniques, such as non-contact tonometry, is utilised to determine ocular behaviour. It is foreseeable that in vivo methods to determine the biomaterial stiffness of the cornea will become increasingly prevalent as they will have significant benefits for tailored vision care procedures. Methods to obtain data with which to derive these properties will include non-contact tonometry which is already used to obtain biomechanical metrics (indicators to biomaterial stiffness), e.g. Bao et al. (2015). The period of loading and unloading of the cornea during non-contact tonometry is in the region of 30–300 ms. This present study demonstrates that the time-dependent effects of loading occur instantaneously and dissipate with periods of relaxation ranging from 1.5 to 150 s. The great majority of work on the accuracy of tonometry methods concentrated on the effect of central corneal thickness on the intraocular pressure measurements. Very few studies considered the effect of material stiffness (e.g. Liu and Roberts 2005), and these only referred to variations in tangent modulus and age. Attention here was limited to noting the effect of variations in tangent modulus and age on IOP measurements by a particular tonometer in each study and relied on clinical data to establish associations. Subsequently, these studies did not consider that tonometers loaded the cornea with different speeds and hence invoked different behaviour patterns due to the inherent viscoelasticity of the tissue. However, from fundamental tests on tissue behaviour, clear viscoelastic responses can be observed, which would be expected to impact the response to various loading conditions, including contact and non-contact tonometry. This proposed model aims to contribute to the advance towards in vivo biomaterial stiffness measurement which is likely to require viscoelastic modelling of the cornea. Verification of results at higher strain rates compatible with non-contact tonometry would be needed before implementation of the viscoelastic model in numerical simulations of non-contact tonometry.

In the absence of in vivo material properties, derivation of material relationships from *ex vivo* experimental studies is required. While there is potential inconsistencies between in vivo and *ex vivo* material behaviour which cannot be ignored, models calibrated from *ex vivo* data have demonstrated the clinical suitability of these methods, for example (Joda et al. 2016).

By addressing a significant gap in viscoelastic representation of the cornea, modelling which includes these properties can now be used to develop and improve treatment procedures based on characteristic behaviour across the population and for an individual patient with material stiffness approximated by their age. In addition, the study has provided valuable information of the magnitude and dissipation periods of viscoelastic effects in the cornea; comibined here with the 3D anisotropic, age-related and regional variations of hyperelastic behaviour, this model is intended to contribute to future in vivo biomechanical analysis.
